# Extracellular Pyrophosphate in the Kidney: How Does It Get There and What Does It Do

**DOI:** 10.1159/000341597

**Published:** 2012-10-12

**Authors:** Shabbir H. Moochhala

**Affiliations:** UCL Centre for Nephrology Royal Free, Royal Free London NHS Foundation Trust, London, UK

**Keywords:** Pyrophosphate, ANKH, ANK, Membrane transport, Efflux, Calcification

## Abstract

Pyrophosphate (PPi) is well known as a regulator of calcification, and the ANKH (ANK in mouse) protein has a role in the membrane transport of PPi. Earlier work concentrated on bones and joints, but ANKH is also likely to have important roles in the kidney, with newer studies focusing on vascular calcification in renal failure. Renal calcification can occur due to a naturally occurring *ANK* mouse mutation, yet other *ANK* mutations do not cause a renal phenotype. Despite evidence over 10 years of ANKH's involvement in PPi transport, efflux of PPi via ANKH has never been demonstrated. Rather than physically moving PPi, the ANKH protein may assist its membrane transport in other ways such as by hydrolysis and compartmentalisation. Protein complexes may account for effects of ANKH that are specific to particular tissues. In the kidney, recent localisation data may be helpful in suggesting physiological roles for ANKH, such as its co-localisation with aquaporin-2 and cilial proteins. Such diverse functions would reflect the ubiquitous nature of ANKH in tissues and its profound evolutionary conservation.

## History of Pyrophosphate as a Crystallisation Inhibitor

In the 1960s, Fleisch and Bisaz [1] deduced the presence of an inhibitory substance in the plasma which prevented mineralisation of collagen. This substance, pyrophosphate (PPi), was able to inhibit crystallisation at 10^–5^m [2], leading Fleisch and Bisaz [1], in 1962, to suggest PPi as a possible therapy for aberrant calcification. PPi binds to the surface of hydroxyapatite (calcium phosphate) crystals, directly inhibiting their growth. Studies over the next 20 years focused on the regulation of extracellular PPi metabolism in bones and joints and measurement of PPi levels in body fluids, but important questions remained unanswered. For example, although low PPi levels were found in the urine of kidney stone formers, it is unclear whether low PPi is a cause or an effect of stone formation. In addition, the finding that the concentration of PPi in urine was greater than that in plasma did not fully explain the origin of urinary PPi, only suggesting that it was somehow either secreted into the tubule or locally generated.

After 1980, surprisingly little further work was performed in this area until Ho et al. [3] cloned the *ANK* gene in 2000 (the nomenclature *ANK* is now used almost interchangeably with the human variant *ANKH*). This re-ignited interest in the mechanism by which PPi levels are regulated and the possible role of the ANKH protein in PPi membrane flux. Ho et al. [3] demonstrated differences in PPi concentrations between fibroblast cell cytoplasm and medium resulting from ANK transfection that were consistent with PPi export. Single gene disorders causing increased or decreased ANKH function have been found to cause specific diseases of under-/over-calcification, respectively, in mice and humans (summarised in table 1), although these do not shed light on the mechanism of ANKH function. With the advent of bisphosphonates, which are stable structural analogues of PPi, as a treatment for bone and mineral diseases, there has been renewed interest in the therapeutic potential of the simple, potent, naturally occurring molecule PPi itself.

## Relevance of PPi and ANKH to Renal Calcification

Abnormalities in extracellular PPi regulation may have possible effects in two areas, namely abnormal calcification of the kidneys (nephrocalcinosis) and calcium-containing renal stone disease. However, in contrast to bone, the tubular environment is highly variable in ionic content over short time periods and is further complicated by the various reabsorptive mechanisms, which themselves can be affected by negative feedback from the tubular contents. This may make measurements of urinary PPi less helpful in elucidating physiological mechanisms. Further confounders include the low absolute levels of PPi and the multi-stage enzymatic assay process used in most studies until recently to estimate PPi concentrations, which have not always reliably excluded the effect of co-existing ATP and orthophosphates. Recently, a zinc-containing organic PPi sensor molecule was developed, which fluoresces in direct proportion to the PPi concentration [4].

The pathophysiology of aberrant calcification in bones/joints due to disorders of the *ANKH* gene has been widely discussed since 2000. Ho et al. [3] found that *ANK* mRNA is expressed not only in joints but also in kidney, and they demonstrated that the autosomal recessive mutant ‘progressive ankylosis’ mouse (hence the name ‘ANK’) exhibits nephrocalcinosis. As in the case of the skeleton, the kidney has an important need to regulate crystal formation in the face of a crystal-inducing environment. Indeed, our group has demonstrated that cells expressing *ANKH* exist in the distal tubule/cortical collecting duct of mouse and human kidney, suggesting that extracellular PPi has a significant role in preventing calcification in this part of the tubule. However, elsewhere in the kidney, ANKH may be predominantly intracellular as is seen in *ANKH*-transfected renal cells [5].

The autosomal recessive mutation *ANKH*^L244S/L244S^, which was recently discovered in a consanguineous family, also causes pathological calcification [6], suggesting that this is an inactivating mutation. However, there was no renal calcification even in severely affected individuals. This may be explained by the existence of other mechanisms to compensate for the effect of lack of PPi in the kidney (e.g. non-ANKH-mediated extracellular PPi generation or other tubular calcification inhibitors), in the same way that ANK-mutant mice do not exhibit vascular calcification despite developing fatally progressive arthritis [7]. Alternatively, it may be that ANK-mediated generation of extracellular PPi is not an important mechanism in the prevention of calcification in the kidney.

## Can PPi Prevent Extra-Renal Calcification in Renal Failure?

Vascular calcification is a major cause of mortality in renal failure. In 2011, an association was found between low PPi levels (approx. 3 µm) and vascular calcification in chronic kidney disease and end-stage renal disease, suggesting extracellular sources of PPi production and hydrolysis [8]. For example, nucleotide pyrophosphatase phosphodiesterase-1 (NPP1) is an ectoenzyme that catalyses the hydrolysis of purine nucleotides, generating PPi. The finding that NPP1 deficiency contributes to rapid arterial calcification in humans [9] supports an important role for the extracellular effects of PPi in tissues other than bone. In important work last year by O'Neill et al. [10], it was found that intraperitoneal injection of PPi in a uraemic rat model reduced the degree of calcification of rat aortas by 70% without affecting certain parameters of bone formation and resorption. However, the treatment did not decrease the overall incidence of aortic calcification, and calcification of smaller vessels was not measured. Significant variability was observed in baseline calcification in the adenine model of renal failure that was used, mirroring the variable situation in human renal failure. Alternatively, this may imply that bone mineral disorder pathophysiology is disordered in the adenine model of renal failure and/or that the effects of other calcification inhibitors have not been accounted for. It was unfortunate that the daily PPi injections induced peritoneal fibrosis. Notably, supra-physiological doses of PPi were required in this study in order to reduce uraemic calcification. This study builds on previous work in humans showing that bisphosphonate treatment reduced progression of coronary artery calcification and inflammatory markers in patients with end-stage renal disease [11].

## Evidence for ANKH Functioning as a PPi Transporter

Many authors now assume that PPi is exported from cells by transport occurring directly by the ANKH protein. But what evidence is there for this? The extracellular PPi concentration is limited by highly active pyrophosphatase enzymes (alkaline and acid phosphatases). Cytosolic PPi is also low because most intracellular PPi is compartmentalised in organelles, where PPi generation occurs mostly in the mitochondria. PPi is a by-product of about 190 biochemical reactions including the synthesis of DNA, RNA, amino acids, proteins and lipids, and rapid removal of the PPi end product must occur to ensure unidirectional and irreversible reactions. PPi removal could occur in three ways: by hydrolysis via cytoplasmic pyrophosphatase; by PPi compartmentalisation via transport into subcellular compartments, or by export from the cytoplasm via a plasma membrane transporter such as the ANKH protein. Ho et al. [3] demonstrated transport by using only indirect measurements of PPi, necessitating a more direct approach. The same group expressed the mouse ANK protein in *Xenopus* oocytes, so allowing direct measurement of transmembrane flux of radiolabelled ^33^PPi [12]. Unfortunately, technical limitations prevented measurement of the physiologically important efflux (cytoplasm towards external medium). Instead, measurements of ANK-mediated ^33^PPi uptake were made, revealing a limited but saturable uptake (V_max_ was only 180 fmol/oocyte/h; apparent K_m_ of approx. 1.3 µm). ANK therefore shows transporter rather than channel characteristics, which is consistent with the proposed 10- to 12-transmembrane segment secondary protein structure of ANK. However, the mechanism of substrate transport is unknown; ANK may function as a facilitated diffusion uniporter, an exchange transporter or a symporter. Since the transmembrane electrical potential of most cells is −60 mV with respect to medium, export of negatively charged PPi alone would be favoured if ANK was a symmetrical transporter. Confirmation of PPi efflux is required to substantiate the physiological role of ANKH in mediating efflux, but experiments in our laboratory have failed to demonstrate efflux using *Xenopus* oocytes.

Recently, a known loss-of-function M48T mutation in *ANKH* was shown using co-immunoprecipitation to interrupt ANKH's interaction with the luminal phosphate transporter PiT-1 (SLC20), which transports monovalent H_2_PO_4_^–^ [13]. It is possible that ANKH may form a complex with other proteins that are associated with its overall function. As already noted, there is a requirement for compartmentalisation of PPi that is generated and utilised by biochemical reactions, so it is likely that further ANKH-protein partner interactions will exist, especially if PPi generation occurs via an enzyme located close to ANKH. This mechanism would explain why measurements of PPi efflux are technically very difficult to perform.

## Generation of Luminal PPi in the Kidney

**Fig. 1:**
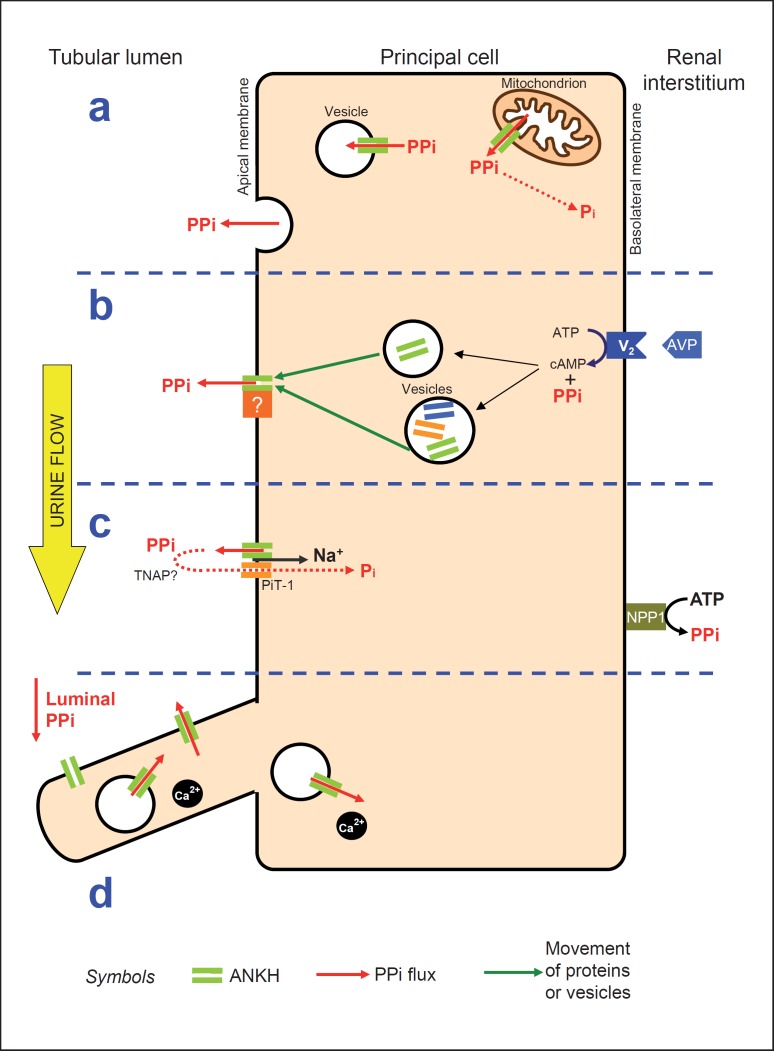
Potential roles of ANKH in the principal cells of the renal collecting duct. **a** Intracellular: PPi is produced in the cytosol as a result of biochemical reactions inside organelles, coupled to ANK in the organelle membrane. Most PPi is rapidly degraded by intracellular pyrophosphatases, but PPi may also be compartmentalised into vesicles containing membrane-bound ANKH. **b** Apical membrane: AVP activates the basolateral V_2_ receptor, generating PPi and stimulating the movement of vesicles containing aquaporin-2 to the apical surface. These vesicles may contain ANKH, or ANKH may exist inside its own vesicles or be chaperoned to the membrane by other proteins. At the apical surface, ANK associates with a partner protein forming a complex which enables PPi export into the lumen. This PPi is either cytosolic or arises as a reaction product of the partner protein. **c** Apical and basolateral membranes: ANK interacts with the sodium phosphate transporter PiT-1 (SLC20A1), allowing apical PPi export into the lumen, with possible recycling of hydrolysed products. On the basolateral membrane, the ectoenzyme NPP1 allows local extracellular generation of PPi. **d** Primary cilium: ANKH present in the cilium and basal body may have a role in sensing PPi already in the lumen. PPi release from intracellular stores could then buffer the calcium fluxes that are induced by cilial deformation. Pi = Orthophosphate.

Immunolocalisation studies in both mouse and human kidney using a specific polyclonal anti-ANKH antibody show ANKH expression restricted mainly to cells in the cortical collecting duct [5]. Co-localisation with aquaporin-2 shows that principal cells express ANKH, but ANKH-positive staining is also seen in intercalated cells. The majority of ANKH localisation is at the apical (luminal) pole of the cells, with some basolateral (interstitial) localisation. Arginine vasopressin (AVP)-induced water reabsorption causes concentration of solutes and increases the risk of precipitation of calcium crystals in the tubule. At this time, the simultaneous secretion of PPi to the cortical collecting duct fluid would be a useful mechanism to reduce the risk of calcification along the collecting duct. Studies using mouse kidney cell lines provide additional evidence that this may occur; we found that AVP stimulates the apical localisation of ANKH in mpkCCD_cl4_ cells, a cortical collecting duct cell model. Localisation studies may suggest new ways in which to investigate the functions of ANKH. We have shown that ANKH localises to the primary cilium and basal body, as well as the apical membrane, of renal epithelial cell lines and murine renal tissue [14]. This places ANKH in a very suitable position from which to sense luminal PPi in the urine (fig. 1).

PPi may also be present in luminal fluid as a result of glomerular filtration or local generation. Indeed, PPi may survive even in the tissue non-specific alkaline phosphatase (TNAP)-rich proximal tubule. Inactivating mutations causing familial arterial and joint calcification were discovered in 2011 in the *NT5E* gene encoding ecto-5′-nucleotidase, which generates adenosine from AMP [15]. Here, decreased adenosine levels may cause a reduction in negative feedback to alkaline phosphatase (TNAP), allowing greater hydrolysis of PPi and hence increased calcification. In the rat nephron, ecto-5′-nucleotidase is found predominantly on the apical aspect of the collecting duct, with some expression in the proximal tubule brush border and distal tubule, but not in the loop of Henle [16]. These varied apical locations support a role for adenosine-induced TNAP inhibition in prolonging the effect of any extracellular PPi present in the tubule, in order to reduce calcification.

## Current Working Hypothesis

There is no doubt that PPi has a key role in regulating calcification in many different tissues, either alone or with other inhibitors. Recently, work has shifted from joint disease to focus on vascular calcification, with very little work on the kidney itself. A component of PPi in the tubular lumen originates from reactions catalysed by the ectoenzyme NPP1, even though the majority of PPi is intracellular. Despite ANKH being implicated in the transport of PPi from the cells to the exterior, the exact physiological function of the ANKH protein has never been clarified. So far, evidence from localisation and the effects of AVP stimulation suggests that ANKH is part of a mechanism which reduces tubular calcification at maximum tubular solute concentration.

Since most PPi metabolism occurs intracellularly, the main role of ANKH may in fact be to allow compartmentalisation of PPi within vesicular endomembranes [5]. Such a role would be of major importance in maintaining the direction of many biosynthetic reactions and may also implicate ANKH as a mechanism of calcium sequestration. The exact mechanisms of ANKH function are likely to be tissue specific and may occur via interactions with other proteins present in that particular tissue. These fundamental biochemical roles are in keeping with the strikingly high degree of evolutionary conservation of the ANKH protein [3] and highlight the likelihood that important additional roles for the protein remain to be defined.

## Disclosure Statement

No conflicts of interest to declare.

## Figures and Tables

**Table 1:** Summary of human and mouse disorders of calcification linked to *ANK* polymorphisms

*ANK* gene activity	Human	Mouse
Increased (hence increased ePPi)	**1. CCAL2** Deposition of calcium pyrophosphate dihydrate crystals causing acute attacks of pseudo-gout in large joints. Mild, late onset. *Autosomal dominant CCAL2:* Gain of function mutations in M48T, E490del and −11CT [17]. *Sporadic CCAL2:* −4 bp G to A SNP (5'UTR) [18]. Demonstrated increased ANK expression and extracellular PPi in vitro. **2. Seizures in autosomal dominant CCAL** Members of one such family experienced early childhood seizures; +4 N-terminal amino acids added due to premature initiation codon, causing likely gain of function [19].	There is no CCAL2 phenotype in mice. *ANK*^M48T/null^ mice showed restored joint function over *ANK*^null/null^ mice but no increased PPi deposition [12].

Decreased (hence decreased ePPi)	**1. CMD** Increased mineralisation causes overgrowth and sclerosis of the craniofacial bones and abnormal modelling of long bone metaphyses, but normal joints. No renal calcification. *Autosomal dominant CMD:* Mutations: F376del (loss), S375del (loss), A380ins (loss) [20]; W292R, C331R, S375del, F377del, P380insA, G389R [21]. *Sporadic CMD:* Complex heterozygous mutation in exon 7 [22]. **2. Autosomal recessive syndrome** Mental retardation, deafness, ankylosis, dental abnormalities. No renal calcification. Homozygous L244S mutation [6].	**1. Progressive ankylosis** Two mouse models featuring severe ankylosis, phenotypically indistinguishable from each other. *ANK*^E440X/E440X^ is a naturally occurring nonsense mutation causing a 53-amino acid C-terminal truncation [3]. *ANK*^null/null^ had a very similar phenotype to *ANK*^E440X/E440X^ [23], except that soft tissue calcification was absent. **2. CMD** C331R and G389R mutations [21] abolish PPi influx in *Xenopus* oocytes and transgenic mice [12], suggesting a dominant negative effect on *ANK*. F377del homozygous knock-in is a mouse model of CMD [24]. **3. Nephrocalcinosis** Increased calcification in the kidneys of adult *ANK*^E440X/E440X^ mice [3].

Polymorphisms or mutations in the *ANK* gene causing either an increase or decrease in gene function exist in both humans and mice and may lead to increased extracellular PPi (ePPi), which reduces calcification, or vice versa. CCAL2 = Chondrocalcinosis type 2; SNP = single-nucleotide polymorphism; UTR = untranslated region; CMD = craniometaphyseal dysplasia.
